# Envelope Correction of Micro-Motion Targets in the Terahertz ISAR Imaging

**DOI:** 10.3390/s18010228

**Published:** 2018-01-15

**Authors:** Qi Yang, Bin Deng, Hongqiang Wang, Yuliang Qin, Ye Zhang

**Affiliations:** College of Electronic Science and Engineering, National University of Defense Technology, Changsha 410073, China; yangqi_nudt@163.com (Q.Y.); oliverwhq@tom.com (H.W.); qinyuliang@nudt.edu.cn (Y.Q.); fighting_zy10@126.com (Y.Z.)

**Keywords:** terahertz radar imaging, envelope correction, micro-motion targets, jump error, drift error

## Abstract

Motion compensation is a crucial step to inverse synthetic aperture radar imaging, and envelope correction is the foundation of motion compensation. Research on envelope correction based on the small-angle imaging model has matured after years of development. However, the small-angle imaging model is not applicable to parameter estimation and imaging of micro-motion targets. According to the characteristics of the micro-motion targets and the superiorities of terahertz imaging radar, an envelope correction method for micro-motion targets in the terahertz region was proposed in this paper, including the jump error correction based on periodic correction and drift error compensation based on nonlinear fitting. Then a 330 GHz imaging radar and two experiments on corner reflectors and a warhead model were introduced. The validity of the method was verified by the experimental results, and the performance of the method was proved by the inverse Radon transform of the range profile sequences.

## 1. Introduction

For non-cooperative space targets, the motion of the targets can be divided into two parts, namely the translational component and rotational component. The translational component will make the high resolution range profile (HPPR) dislocation in the range, and also introduces the additional phase error which will cause defocus in the azimuth [1,2,3]. So translation compensation is the prerequisite and crux to inverse synthetic aperture radar (ISAR) imaging, and will directly affect the quality of the imaging results. In general, translation compensation in the small-angle imaging model consists of envelope correction and phase correction, and envelope correction should be firstly considered. Unlike the small-angle targets, the micro-motion targets, such as the helicopter with rotating rotors and the precession ballistic targets, often require a long observation times for parameter estimation and imaging, and range migration is not inevitable. In this condition, the traditional envelope correction criteria, such as cross correction criterion and minimum entropy criterion, lose efficacy gradually with the decreasing of the correlation coefficient between each range profile. In addition, dechirp-on-receive is often adopted in ISAR system, and the reference range is time varying due to the high-speed movement of the target. The changing reference range will also affect the time delay values of the envelopes and make them disordered over time.

Current research on envelope correction is mainly aimed at small-angle imaging models in the microwave band, and a lot of novel approaches and their improvements have emerged during decades of development [4,5,6]. However, there are few studies about envelope correction of the micro-motion targets in the terahertz band. In view of micro-motion targets, J.Q. Li et al. introduced a novel compensating method for the rotationally symmetric targets based on the micro-Doppler symmetry cancellation effect [7]. However, it is more suitable for synthetic aperture radar processing because the reference range is constant in simulation. W.P. Zhang et al. proposed a frequency estimation method of micro-motion targets with complicated translations based on piecewise translation compensation and time–frequency squared difference sequences [8], which is only applicable to narrowband systems. Beyond that, B. Yuan and X.R. Bai et al. also made a prominent work on micro-motion target imaging [9,10,11], but they mainly focused on signal separation of non-rigid targets or other new imaging methods, and did not involve envelope correction of micro-motion targets. In addition, the current studies are mainly in the microwave band. Compared with the microwave radar systems, terahertz radar systems have an advantage for high resolution imaging because of the large wideband and the short wavelength of the terahertz signal. At the same time, the high range resolution also puts forward higher requirements for envelope correction. In 2016, T. Liu et al. proposed an envelope alignment technique for terahertz radar ISAR imaging of maneuvering targets [12], which is the only literature about envelope correction in the terahertz band. At present, there are no reports and literature of the relevant studies on envelope correction of micro-motion targets in the terahertz ISAR imaging.

Therefore, according to the characteristics of the micro-motion targets and the superiorities of terahertz imaging radar, an envelope correction method for micro-motion targets in the terahertz region is proposed in this paper. The paper is organized as follows: the envelope correction method of micro-motion targets in the terahertz radar imaging is introduced in detail in Section 2. The method mainly includes two aspects: jump error correction based on the periodicity and the drift error compensation based on the sinusoidal characteristic of the HRRP. In Section 3, an experimental radar system with a carrier frequency of 330 GHz is briefly introduced, and experiments on two rotating corner reflectors and a precession warhead model are carried out. The data processing results in Section 3 verifies the efficiency of the method proposed in this paper. Conclusions are presented in the last section.

## 2. Envelope Correction Method

### 2.1. Signal Model of Micro-Motion Targets and Simulation Scenario

The motion model of the micro-motion targets can be simplified to harmonic motion, and the expression of a micro-motion target with K scatterers is as

(1) $$R_{k}=R_{0}+a_{k}\sin(\omega_{k}t_{m}+\varphi_{k}),\;k=1,2,\ldots ,\;K$$

where $R_{0}$ is the initial distance between the radar and the micro-motion target. $a_{k}$, $\omega_{k}$, and $\varphi_{k}$ are the amplitude, angular velocity, and initial phase of the micro-motion scatterer k, respectively. According to the radar echo signal model of micro-motion targets in reference [13], the echo signal of the micro-motion target with K scatterers can be expressed as

(2) $$s_{if}(\hat{t},t_{m})=\sum_{k=1}^{K}rect\left(\frac{\hat{t}-2R_{k}/c}{T_{p}}\right)\exp\left[-j\frac{4\pi }{c}\gamma \left(\hat{t}-\frac{2R_{ref}}{c}\right)\left(R_{k}-R_{ref}(t_{m})\right)\right]$$

where $\hat{t}$ and $t_{m}$ represent the fast-time in range and slow-time in azimuth respectively. $T_{p}$ is the pulse width in the linear frequency modulation (LFM) mode or the frequency-sweep period in the frequency modulated continuous wave (FMCW) mode. $f_{c}$ is the carrier frequency of the radar system, and γ is the chirp rate. c is the speed of light. $R_{ref}$ is the reference range during dechirp receiving, which is time varying in ISAR observation. After that, the range profile can be obtained by the Fourier transform of Equation (2) to the fast-time

(3) $$S_{if}(f_{i},t_{m})=T_{p}\sum_{k=1}^{K}\sin c\left[T_{p}\left(f_{i}+2\frac{\gamma }{c}\left(R_{k}-R_{ref}(t_{m})\right)\right)\right]\cdot \exp\left(-j\frac{4\pi f_{c}}{c}\left(R_{k}-R_{ref}(t_{m})\right)\right)$$

where $\sin c(\alpha )=\frac{\sin(\pi \alpha )}{\pi \alpha }$. It can be seen from Equation (3), the range profile envelope of the micro-motion target is sinusoidal modulation if the reference range is constant during observation, and the modulation period is in accordance with the micro-motion period. However, the sinusoidal modulation is destroyed by the time-varying reference range in the ISAR system, bringing difficulties to parameter estimation and imaging of micro-motion targets.

**Figure 1:**
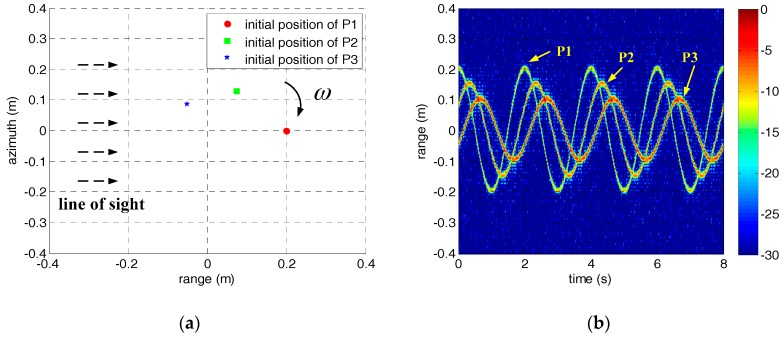
The simulation scenario and the HRRP of the rotating scatterers. (**a**) The initial position of the scatterers. (**b**) The HRRP of the scatterers.

In order to illustrate the envelope correction method clearly, a simulation scenario consisting of three rotating scatterers is established, as shown in Figure 1a. The rotating radii of the scatterers are 0.2, 0.15, and 0.1 m, respectively. The normalized scattering intensities are set to 0.6, 0.8, and 1. The rotating angular velocity ω is π rad/s, and the observation time lasts 8 s in simulation. The pulse repetition frequency (PRF) of the signal is set to 1000. The HRRP of the rotating scatterers when the signal-to-noise ratio SNR = 0 dB is shown in Figure 1b. The sinusoidal modulation of the range profile is obvious because the reference range is constant during simulation. In practice, the range profiles will be disorderly with the change of the reference range. In other words, the HRRP in Figure 1b is a typical result of a micro-motion target after envelope correction, and the characteristic analysis is necessary if we want to realize envelope correction.

### 2.2. Jump Error Correction Method Based on the Periodicity of the HRRP

The traditional envelope correction methods in small-angle models are mainly based on the correlation among range profiles with the assumption that migration through resolution cells (MTRC) does not take place during a very short time. Under this assumption, the scatterers within each resolution cell are fixed, and the shapes of each range profile are very similar. Although this assumption is not suitable for micro-motion targets in the terahertz band, the similarity of the adjacent range profiles remains at a high level. Therefore, the classical envelope correction method based on adjacent correlation in ISAR processing still has a certain effect for micro-motion targets. The core operation of adjacent correlation method is the cyclic shift of each range profile and the cyclic shift values can be obtained as

(4) $$\overset{⌢}{\xi }=\arg\left[\underset{i}{\max}\left({\left(S_{n}^{i}\right)}_{abs}\cdot {\left(S_{n}^{ref}\right)}_{abs}\right)\right]$$

where $\overset{⌢}{\xi }$ is the cyclic shift value based on the adjacent correlation method. When dealing with the n-th range profile $S_{n}$, the reference signal $S_{n}^{ref}$ is the previous range profile of $S_{n}$, that is

(5) $$S_{n}^{ref}=S_{n-1}$$

**Figure 2:**
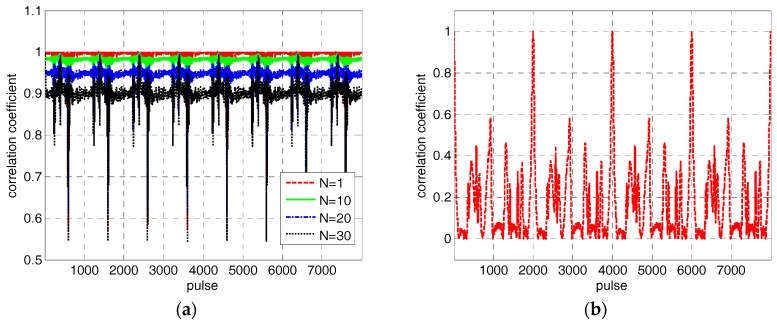
The correlation coefficients of the simulation data. (**a**) The correlation coefficients at different accumulation numbers. (**b**) The corrcetion coefficients between the first range profile and others.

However, there are two problems in this method: one is jump error and the other is drift error. Although we can obtain the cyclic shift values based on adjacent correlation method, the tiny error is there and it will deteriorate into big shift error through accumulation of many range profiles. In addition, the similarity of the adjacent range profiles will be destroyed and jump error occurs if several range profiles are abnormal. The features of jump error and drift error on the range profile sequence are the ‘sudden jump’ phenomenon and the ‘integral decline’ phenomenon, respectively. To overcome the jump error and drift error, the accumulation correlation method is often adopted in practice. The reference signal in the accumulation correlation method is a weighted sum of several aligned range profiles, and two typical weighting forms are

(6) $$\left\{ \begin{array}{ll} S_{n}^{ref}=\frac{1}{N}\sum_{n-N}^{n-1}S_{i}, & \mathrm{rectangular}\;\mathrm{window}\;\mathrm{weighting}\\ S_{n}^{ref}=\sum_{n-N}^{n-1}{\left(\frac{1}{2}\right)}^{i-n+2}S_{i}, & \mathrm{exponential}\;\mathrm{weighting} \end{array}\right.$$

where N is the accumulation number of range profiles in the accumulation correlation method. However, this method is clearly not suitable for micro-motion targets because the correlation coefficients between each range profile decline rapidly with the increase of the accumulation number. The underlying reason is that the shapes of the range profiles change rapidly because of micro-motion. We draw the correlation coefficient curves of the HRRP in Figure 1b when N is 1, 10, 20, and 30, respectively, as shown in Figure 2a. The accumulation correlation method will be degenerated to adjacent correlation method automatically when N equals 1. Although correlation coefficients are relatively large when N=1, the adjacent correlation method has no resistance to the jump error and drift error as stated earlier. Conversely, a larger accumulation number N may help to reduce and even eliminate the jump error and drift error, but the performance of envelope correction will degrade a lot because of the low correlation coefficients. Therefore, we have to choose a small accumulation number in practice to ensure the overall performance of envelope correction firstly, and search for other ways to compensate the jump error and drift error in the meantime.

Due to the periodicity of micro-motion, the power of the range profile sequence of the micro-motion target is still periodic, even though it is disordered in range because of the changing of the reference range. It means that we can estimate the micro-motion period T from the power of the range profile sequence before envelope correction by some mature methods, such as autocorrelation operation, spectrum analysis, and cepstrum analysis [14,15]. According to the characteristics of micro-motion targets, a jump error correction method based on the periodicity of the HRRP is proposed in this paper. Take the simulation result in Figure 1 for example again, the correlation coefficient curve between the first range profile and others is shown in Figure 2b. It is obvious that the correlation coefficient reaches its maximal value periodically. In other words, range profiles at the same location in each micro-motion period are very similar, even though they change rapidly because of micro-motion. Therefore, we can correct the jump error based on the periodicity of the HRRP after a rough correction based on the adjacent correlation method. The reference signal is set to the weighted sum of range profiles of other periods that sharing the same location with the current range profile, as follows.

(7) $$S_{n}^{ref}=\frac{S_{n-T\cdot PRF}+S_{n-2T\cdot PRF}+\cdot \cdot \cdot +S_{n-KT\cdot PRF}}{K}$$

where K is the accumulation number of periods. The weighting method is rectangular window weighting in Equation (7). The method relies on the periodicity of the HRRP and corrects jump error by the weighted sum of several range profiles from different periods. To prevent the jump error in the first few periods of observation, a reverse operation from the last range profile is necessary at times. With the guarantee of overall performance, the method can correct the jump error effectively. However, drift error still remains and the range profile sequence maybe inclined.

### 2.3. Drift Error Compensation Method Based on the Sinusoidal Characteristic of the HRRP

After jump error correction, the range profile sequence of the micro-motion targets is continuous, but it might be declining because of the drift error. As mentioned above, the core operation of envelope correction is cyclic shift, which will not change the shapes of the range profiles. In this situation, the whole range profile sequence will be corrected automatically if we can correct one of the micro-motion scatterers. Therefore, the first thing to do is extracting the range what we call it the initial drift range r(t) of a micro-motion scatterer. There are many ways to extract the initial drift range, and the Viterbi algorithm which was usually used for instantaneous frequency extraction in the time-frequency domain is a robust way. The Viterbi algorithm was proposed by G.D. Forney in 1967, and has been widely applied to many occasions [16,17].

The initial drift range is mainly composed of two parts: one is the sinusoidal range resulting from micro-motion, and the other is the drift range caused by drift error. Only the drift range is obtained exactly can drift error be compensated well. In this paper, a drift error compensation method based on the sinusoidal characteristic of the HRRP is proposed. It is on the basis that the range profile sequence of the micro-motion targets is sinusoidal modulation and the modulation period equals the micro-motion period. On this basis, we can fit the initial drift range by a nonlinear model as

(8) $$\overset{⌢}{r}(t)=a\sin\left(\frac{2\pi }{T}t+b\right)+c+dt+et^{2}$$

where $\overset{⌢}{r}(t)$ is the fitting result what we call nonlinear fitting range. The sinusoidal part in the nonlinear fitting range presents the micro-motion of the scatterrers, and the quadratic polynomial part is used for fitting the drift range since it is slowly-varying. a, b, c, d, and e are the parameters which can be obtained by nonlinear fitting. After this, the drift range, or the fine compensation range in the drift error compensation can be expressed as

(9) $$\Delta y(t)=r(t)-\left[a\sin\left(\frac{2\pi }{T}t+b\right)+c\right]$$

**Figure 3:**
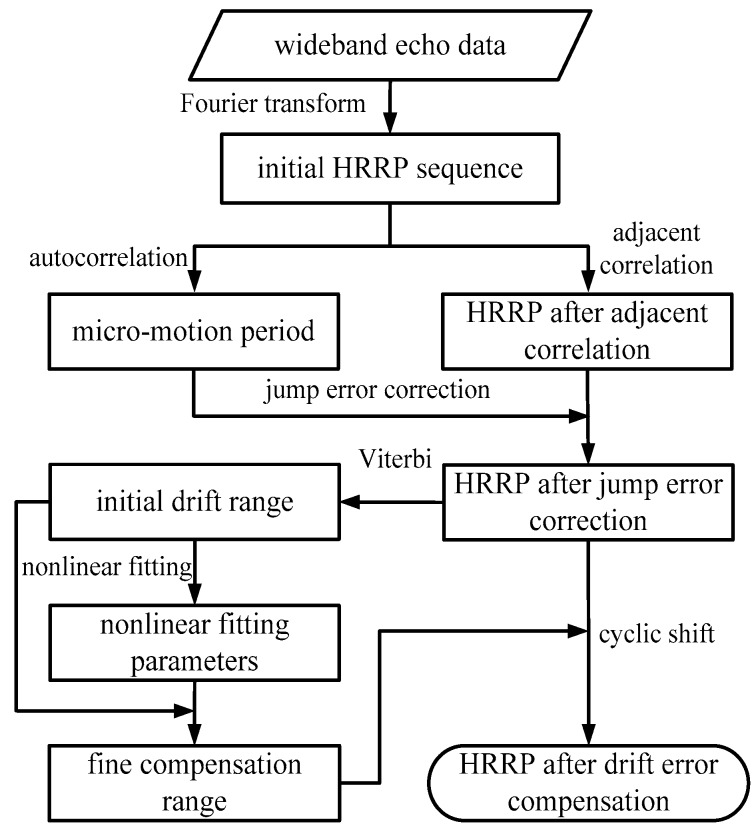
Flow diagram of the envelope correction method.

Through the secondary compensation by the fine compensation range, the range profile sequence of the micro-motion target is sinusoidal over time. At this point, other operations such as parameter estimation and imaging can be carried out smoothly. The flow diagram of the method is shown in Figure 3.

## 3. Experiments and Data Processing Results

### 3.1. Terahertz Radar System and Experiments

**Figure 4:**
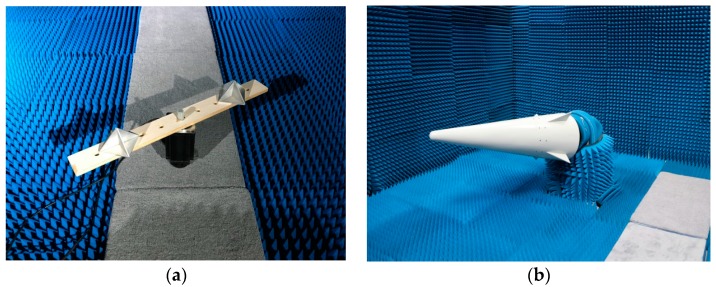
The micro-motion targets in experiments. (**a**) The rotating corner reflectors. (**b**) The precession warhead model.

In order to verify the envelope correction method, a terahertz imaging radar system is built and experiments on micro-motion targets are carried out. The terahertz radar system is based on the LFM pulse principle and has a 330 GHz of central frequency with a synthetic bandwidth of 10 GHz, thereby realizing a 1.5 cm theoretical range resolution. The terahertz signal is transmitted by a cone-shaped horn antenna after 24 times frequency multiplication of a Ku band sweeping generator in the transmitting chain, and the transmitting power is greater than 5 mW. The PRF of the transmitting signal is 1000, and the sample number of each pulse is 2048. In order to verify the envelope correction method, we designed two experiments in this paper. Experiment 1 is on two rotating corner reflectors driving by a motor, as shown in Figure 4a. The rotating radii are 0.18 m and 0.32 m, respectively. The rotating angle velocity is set to π/2 rad/s, corresponding to the micro-motion period of $T_{1}=4$ s. Experiment 2 is on a precession warhead model, and the precession angle velocity is π rad/s, corresponding to the micro-motion period of $T_{2}=2$ s (Figure 4b). The radar system and the warhead model are placed in an absorbing chamber during the experiment in order to reduce the impact of the background noise.

### 3.2. Experimental Results

**Figure 5:**
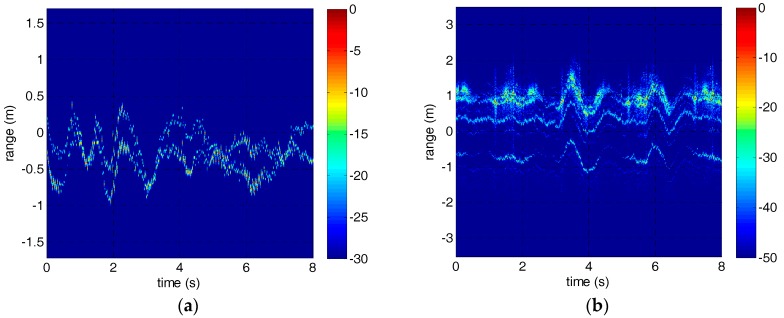
The initial HRRPs of the micro-motion targets. (**a**) The HRRP of the corner reflectors. (**b**) The HRRP of the warhead model.

Through dechirp receiving, the terahertz echo signal is down conversed to baseband and transmitted to the PC for further processing after I/Q demodulator and A/D sampling. The HRRPs in two experiments are shown in Figure 5. Obviously, the HRRPs are disordered due to the adjustment of the reference range and the motion of the targets.

**Figure 6:**
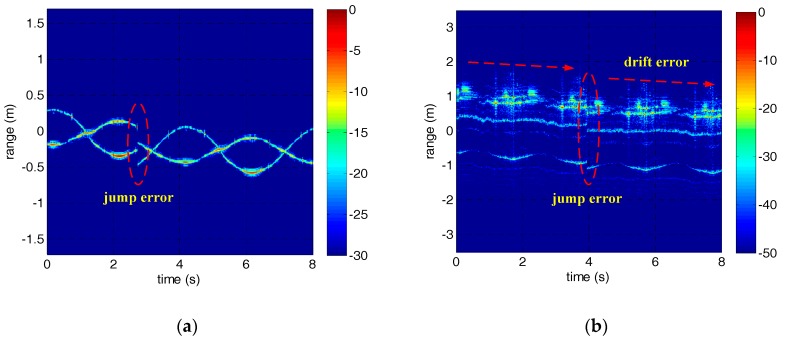
The HRRPs of the micro-motion targets after adjacent correlation correction. (**a**) The HRRP of the corner reflectors. (**b**) The HRRP of the warhead model.

According to the adjacent correlation correction principle mentioned in Section 2, the rough correction results of the micro-motion targets are shown in Figure 6. However, due to the inherent defects of this method, jump error has appeared in both experiments. In addition, there is also drift error in the HRRP of the warhead model.

**Figure 7:**
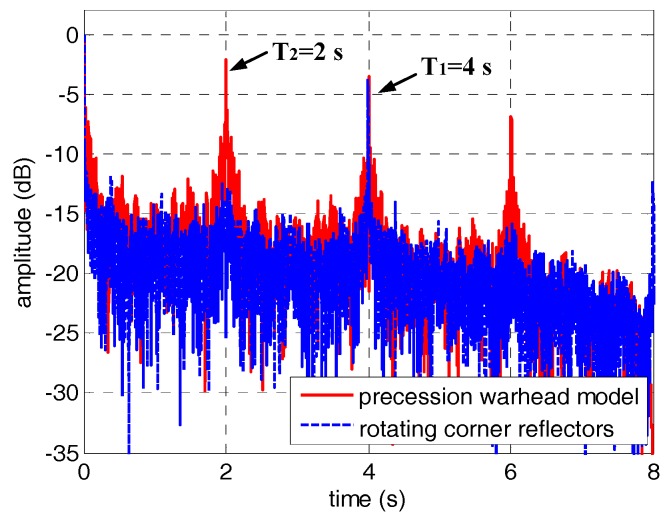
The estimation results of micro-motion period based on autocorrelation.

**Figure 8:**
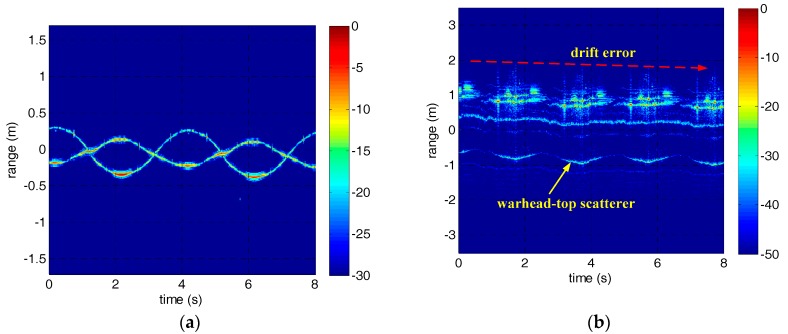
The HRRPs of the micro-motion targets after jump error correction. (**a**) The HRRP of the corner reflectors. (**b**) The HRRP of the warhead model.

In order to correct the jump error, we applied the jump error correction method based on the periodicity of the HRRP to Figure 6. Since this method is heavily dependent on the estimation of micro-motion period, the estimation results of micro-motion period based on autocorrelation were shown firstly in Figure 7. As can be seen from Figure 7, the micro-motion period can be estimated accurately before envelope correction, which is the foundation of jump error correction. The estimated micro-motion periods of the corner reflectors and the warhead model are 4 s and 2 s, which are in good agreement with the settings. The HRRPs of the micro-motion targets after jump error correction are shown in Figure 8. At this point, the jump error has been well corrected and the HRRP sequences show their continuity. However, drift error in experiment 2 still remains to be compensated.

**Figure 9:**
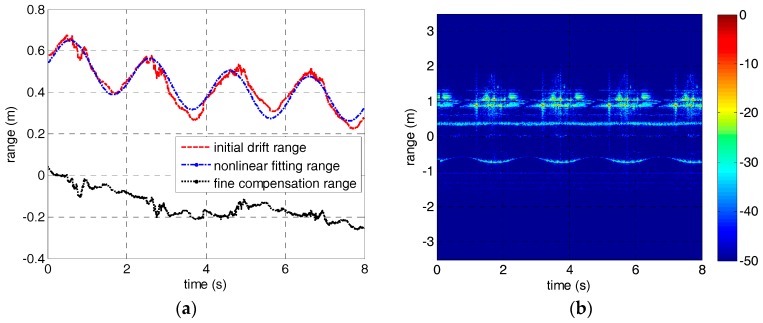
The nonlinear fitting result and the HRRP of the precession warhead model after drift error correction. (**a**) The nonlinear fitting result. (**b**) The HRRP of the warhead model.

As for drift error in experiment 2, we extracted the initial drift range of the warhead-top scatterer by Viterbi algorithm and fitted it using the nonlinear fitting model discussed in Section 2. Then the fine compensation range was obtained according to the fitting parameters, and has been carried on drift error compensation. The nonlinear fitting result and the HRRP of the precession warhead model after drift error compensation are shown in Figure 9. It can be seen from Figure 9b that the drift error has been well compensated. The results verify the efficiency of the method proposed in this paper.

### 3.3. Performance Analysis of the Method

Previous research investigations on imaging of micro-motion targets are partly based on echo separation of the micro-motion part and the rigid part. In this case, envelope correction can be realized relying on the range profile of the rigid part. The other part treated the micro-motion part as interference, and focused on micro-motion suppression. Therefore, there are few studies about envelope correction of the micro-motion targets at present, and the methods in reference [7,8] are not applicable to our situation. In essence, the method in this paper is an improvement of the adjacent correlation method in ISAR imaging. An effective improvement was proposed to make it suitable for micro-motion targets. So we compared the proposed method with the traditional adjacent correlation method in this section.

Until now, research on envelope correction of micro-motion targets is quite limited, not to speak of the evaluation standard. Therefore, in order to analyze the performance of our method, we put forward an evaluation standard based on inverse Radon transform (IRT) according to the sinusoidal modulation characteristics of the micro-motion targets. IRT is a classical approach to curve detection, and has been widely used in radar applications, such as parameter estimation and imaging of micro-motion targets. It can map sinusoidal curves to peaks in parameter space according to the image reconstruction theory, and the locations and the focusing performance of the peaks are directly associated with the characteristics of the sinusoidal curves [18]. If there is a sinusoidal curve in the center of the input image, the image can be represented by the 2D form

(10) $$\hat{g}(\rho ,\theta )=\delta \left[\rho -\alpha \sin(\theta +\varphi )\right]$$

where θ and ρ stand for the horizontal and vertical axis of the input image, respectively. α and φ is the amplitude and the initial phase of the sinusoidal curve. The image after IRT may be written as shown below according to the Fourier slice theorem.

(11) $$\begin{array}{ll} g(x,y) & =\int_{-\infty }^{\infty }\int_{-\infty }^{\infty }\int_{-\infty }^{\infty }\hat{g}(\rho ,\theta )e^{-j2\pi \rho v}e^{j2\pi (k_{x}x+k_{y}y)}d\rho dk_{x}dk_{y}\\ & =\int_{-\infty }^{\infty }\int_{-\infty }^{\infty }\int_{-\infty }^{\infty }\delta (\rho -A\sin(\theta +\varphi ))e^{-j2\pi \rho v}d\rho \cdot e^{j2\pi (k_{x}x+k_{y}y)}dk_{x}dk_{y}\\ & =\int_{-\infty }^{\infty }\int_{-\infty }^{\infty }e^{-j2\pi vA\sin(\theta +\varphi )}\cdot e^{j2\pi (k_{x}x+k_{y}y)}dk_{x}dk_{y}\\ & =\int_{-\infty }^{\infty }\int_{-\infty }^{\infty }e^{-j2\pi Ak_{x}\sin\varphi }\cdot e^{-j2\pi Ak_{y}\cos\varphi }\cdot e^{j2\pi (k_{x}x+k_{y}y)}dk_{x}dk_{y}\\ & =\int_{-\infty }^{\infty }e^{j2\pi (x-A\sin\varphi )k_{x}}dk_{x}\cdot \int_{-\infty }^{\infty }e^{j2\pi (y-A\cos\varphi )k_{y}}dk_{y}\\ & =\delta (x-\alpha \sin\varphi )\delta (y+\alpha \cos\varphi ) \end{array}$$

where, $k_{x}=v\cos\theta$, $k_{y}=v\sin\theta$. It is obvious that the sinusoidal curve ρ=αcos(θ+φ) in the input image is mapped to a point (αsinφ,αcosφ) in parameter space by IRT. In addition, the parameters of the sinusoidal curve have the following relationship with the location of the peak.

(12) $$\left\{ \begin{array}{l} \alpha =\sqrt{{\left(\alpha \sin\varphi \right)}^{2}+{\left(\alpha \cos\varphi \right)}^{2}}\\ \varphi =\arctan\left(\frac{\alpha \sin\varphi }{\alpha \cos\varphi }\right) \end{array}\right.$$

**Figure 10:**
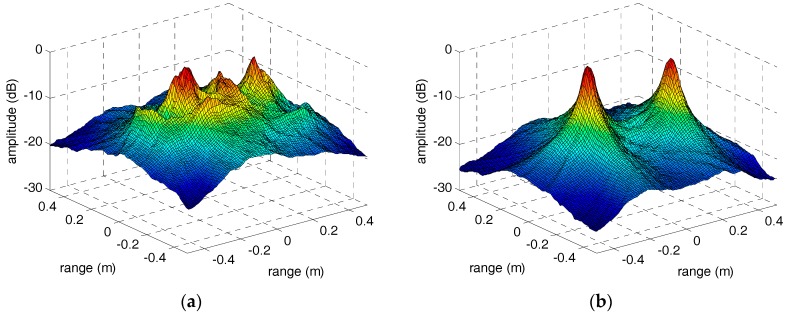
The IRT results of the corner reflectors’ HRRP sequences through adjacent correlation correction and our method. (**a**) The adjacent correlation correction. (**b**) The method in this paper.

**Figure 11:**
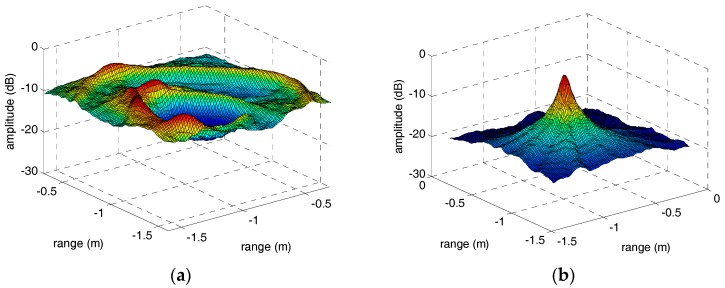
The IRT results of the warhead model’s HRRP sequences through adjacent correlation correction and our method. (**a**) The adjacent correlation correction. (**b**) The method in this paper.

**Figure 12:**
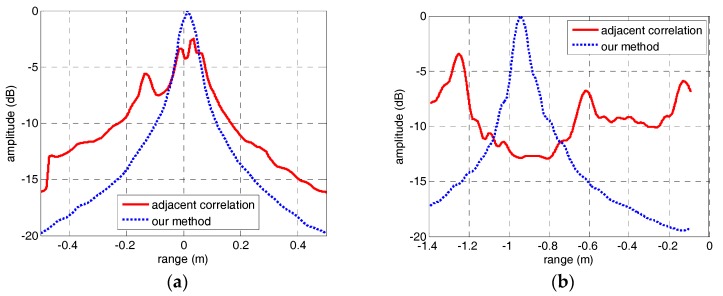
Comparison of the focusing performance of the IRT results through adjacent correlation correction and the method in this paper. (**a**) The adjacent correlation correction. (**b**) The method in this paper.

However, when the sinusoidal curves are discontinuous or declining, the focusing performance of peaks in parameter space will deteriorate severely. In other words, we can treat the focusing performance of peaks in parameter space as an evaluation standard of envelope correction method of micro-motion targets. Therefore, in order to compare the performance of the adjacent correlation method and our method in this paper, the IRT results of the HRRP sequences through adjacent correlation method and our method are shown in Figure 10 and Figure 11. In Figure 10a, the peaks corresponding to the two micro-motion corner reflectors did not focus well compared with Figure 10b because the HRRP sequence in Figure 6a is discontinuous. The sinusoidal fragment which is not in the center of the input image has no contribution to the integration operation during IRT. In Figure 11a, the IRT result of the HRRP sequence in Figure 6b is completely defocusing because of the drift error and the jump error. However, after correction by our method, the sinusoidal trajectory of the warhead top is very clear and its IRT result in Figure 11b focuses well. The IRT results of the HRRP sequences after envelope correction by our method demonstrate the excellent performance of the method proposed in this paper. Moreover, for the quantitative evaluation of the methods, the section curves of peaks in Figure 10 and Figure 11 are presented in Figure 12. Comparison of the section curves proves again that the impact of jump error and drift error in the adjacent correlation method is serious, and it can be overcome by the method in this paper.

## 4. Conclusions

For micro-motion targets in the terahertz ISAR applications, an envelope correction method was proposed in this paper. Unlike the traditional envelope correction methods in a small-angle model, our method can adjust the range profile sequence and make it sinusoidal over time. According to the characteristics of the micro-motion targets, the basic operation adopted in the method is adjacent correlation criterion, even though it has two inherent defects—jump error and drift error. Therefore, after envelope correction based on adjacent correlation, a jump error correction method based on the periodicity of the HRRP was proposed. It takes full advantage of the similarity of range profiles at the same location of each micro-motion period, and takes the weighted sum of range profiles of other periods that sharing the same location with the current range profile as the reference signal. As for drift error, we proposed a correction method based on the sinusoidal characteristic of the HRRP. The parameters of the initial drift range can be fitting by a nonlinear model, and the fine compensation range can be deduced by the initial drift range and its fitting parameters. Finally, we built a 330 GHz imaging radar system and carried out two experiments on rotating corner reflectors and a precession warhead model, respectively. The experimental results verify the validity of the method. However, this method is not suitable for real-time processing because the nonlinear fitting relies on the global property of the range profile sequence, and the real-time envelope correction method for micro-motion targets in the terahertz ISAR imaging still need further research.
